# Global disparities in regulatory approval of prophylactic vaccines, 2005–2024

**DOI:** 10.7189/jogh.16.04145

**Published:** 2026-07-10

**Authors:** Rizhen Wang, Jing Chen, Yangmu Huang, Ming Xu

**Affiliations:** 1 Department of Global Health, School of Public Health, Peking University, Beijing, China; 2 Department of Pharmacy Administration and Clinical Pharmacy, School of Pharmaceutical Sciences, Peking University, Beijing, China; 3 International Research Centre for Medicinal Administration, Peking University, Beijing, China; 4 Institute for Global Health and Development, Peking University, Beijing, China

## Abstract

**Background:**

Timely approval of prophylactic vaccines is essential to equitable access, especially in low- and middle-income countries (LMICs) disproportionately affected by vaccine-preventable diseases. In this cross-sectional analysis, we characterised the global landscape of regulatory approvals for new prophylactic vaccines over the past two decades.

**Methods:**

We retrieved data from Pharmaprojects for new prophylactic vaccines approved globally from 2005–2024. We quantified approval volume and delay – defined as the number of countries, territories, and areas (CTAs) approving each vaccine, and the time (in days) between global first approval and subsequent national approvals. We conducted an exploratory multivariable linear regression analysis to identify factors potentially associated with approval delays.

**Results:**

Of the 416 prophylactic vaccines, 67.8% received approval in only one CTA, and just 12.8% reached more than five CTAs between 2005 and 2024. By 2024, the European region had the highest total number of approvals (n = 2410), while Africa had the fewest (n = 56). Median global approval delay for prophylactic vaccines was 213 days (interquartile range (IQR) = 93.0, 768.8), ranging from 161.0 days (IQR = 84.0, 646.0) in Europe to 705.5 days (IQR = 183.3, 2220.3) in the Western Pacific region. Our multivariable models showed that a lower income level may be associated with longer delay. Other factors, such as the type of vaccine and regulatory designation, were also identified as potential correlates.

**Conclusions:**

Disparities in vaccine approval volume and timing persist across regions. Targeted policy actions are needed to strengthen regulatory systems in LMICs and reduce structural barriers to equitable vaccine access globally.

Despite vaccines saving millions of lives, major disparities in the approval of new prophylactic vaccines across countries hinder their development and availability. Low-and middle-income countries (LMICs) – where these vaccine-preventable diseases (VPDs) are most burdensome – often wait years or even decades after new vaccines are introduced in high-income settings [1,2].

To address these inequities, international initiatives, particularly those led by the World Health Organization (WHO), United Nations International Children’s Fund (UNICEF), and the Global Alliance for Vaccines and Immunisation (Gavi), have attempted to accelerate vaccine access in LMICs [2]. WHO’s prequalification mechanism ensures that vaccines used in immunisation programmes are safe and effective [2]. UNICEF relies on WHO prequalification to guide its procurement decisions [1], while Gavi provides financial support to help the world’s poorest countries access these new and underused vaccines [3]. Additionally, in an effort to accelerate vaccine availability in response to future pandemic threats, the Coalition for Epidemic Preparedness Innovations has articulated an ambitious goal – vaccines should be ready for initial authorisation and scaled-up manufacturing within 100 days after the identification of a pandemic-causing pathogen, where feasible and appropriate [4].

Despite multiple mechanisms shaping the availability of vaccines in different settings, regulatory approval represents the earliest measurable milestone in the pathway toward vaccine availability [5]. Although approval alone does not ensure procurement, distribution, or population-level uptake, it constitutes the regulatory gateway through which all subsequent access mechanisms must pass [6,7]. Delayed or absent approval in LMICs may restrict procurement options and prolong vaccination gaps even when vaccines are already available elsewhere [6]. For this reason, the timing of regulatory approval provides an important upstream, internationally comparable indicator of potential inequities in vaccine availability.

A recent study reported a median (MD) delay of 5.4 years between first licensure and vaccine introduction in Gavi-supported countries, and the delays were experienced regardless of whether a vaccine was ‘older’ or more recently supported by Gavi [8]. Delays in approval of new prophylactic vaccines are not exclusive to LMICs – for instance, Japan approved its first COVID-19 vaccine several months after other high-income countries (HICs) [9]. Although previous studies have examined vaccine approval timelines in selected countries or for specific vaccine groups, the global landscape of regulatory approval inequities remains poorly characterised. In particular, little is known about how approval delays vary systematically across WHO regions and World Bank income groups over time [8,10]. This gap limits global health actors’ ability to identify structural bottlenecks in regulatory pathways that may contribute to inequitable vaccine availability.

In this study, we systematically identified global commercially developed prophylactic vaccines from 2005–2024, which constitute a substantial proportion of the total prophylactic vaccines. We characterised approval numbers and delays for prophylactic vaccines by WHO region and World Bank income group. Additionally, we conducted an exploratory investigation into factors associated with vaccine approval delays that may inform future targeted policy interventions.

## METHODS

### Data source

Since regulatory approval is a mandatory prerequisite for vaccine availability – representing a measurable, internationally comparable milestone that directly precedes all downstream access pathways – we defined vaccine availability as the regulatory approval of a prophylactic vaccine within a given country [7,11]. Information on new prophylactic vaccine regulatory approvals was derived from Pharmaprojects [12], a commercial database that tracks the development and registration of pharmaceutical products using public and proprietary sources (Figure S1 in the **Online Supplementary Document**). Commercially developed vaccines represent the predominant share of globally approved prophylactic vaccines [13]. Pharmaprojects systematically tracks vaccines developed by pharmaceutical companies, while regulatory submission data for publicly developed vaccines are dispersed across national regulatory authorities (NRAs) without a standardised structure [14]. Because Pharmaprojects relies largely on voluntary reporting by manufacturers, data on approval dates may be incomplete, particularly for LMICs. In the absence of approval dates, we used the recorded launch date in Pharmaprojects as a proxy for vaccine availability [15]. Other commercial and public data sources, including the Yaozhi database [16], and the COVID-19 Vaccine Tracker Database [17], as well as regulatory announcements, published literature, and pharmaceutical companies’ websites, were used to cross-check the approval-date information provided by Pharmaprojects. Discrepancies were resolved by prioritising official regulatory sources and, where applicable, verifying extracted variables through dual review. Vaccines were excluded if they were duplicates, lacked an approval date, were approved outside the 2005–2024 period, or if the information could not be cross-checked. We used the STROBE guidelines for cross-sectional studies for reporting [18,19].

### Data extraction

Using the anatomical therapeutic chemical classification system in Pharmaprojects, we selected all vaccines whose therapeutic class included ‘prophylactic’ as prophylactic candidates. In Pharmaprojects, new prophylactic vaccines were defined inclusively as vaccines with a different brand name, including vaccines with updated formulations or strain modifications. We focused on vaccines receiving their first regulatory approval from 2005 onwards for two reasons. First, systematic tracking of global vaccine regulatory approvals in databases such as Pharmaprojects became substantially more complete after the early 2000s. Second, the period after 2005 captures major transformations in the global vaccine innovation landscape, including the emergence of novel vaccine platforms and accelerated development timelines [20]. Centralised marketing authorisation by the European Medicines Agency confers simultaneous approval across all European Union member states, Iceland, Liechtenstein, and Norway; treating such approvals as equivalent to approval in each participating country is consistent with prior studies [21–23]. We coded vaccine type, valence, indication, delivery route, and regulatory status at the vaccine level. We classified vaccines as whole-pathogen, split-virus, subunit, nucleic acid, or ‘multiple’ if they combined multiple technologies, following the classification proposed by MacPherson et al. [24]. We defined a combination vaccine as one that contained antigens from either different strains of a single pathogen (multivalent vaccines) or from multiple pathogens (multipathogen vaccines) [25]. We identified the lead indication for vaccines targeting a single pathogen, as reported by Pharmaprojects. Combination vaccines with multiple indications were labelled as ‘multiple diseases.’ Delivery routes were grouped into injectable and novel routes, with the latter including oral, inhaled, or transdermal delivery. A label of ‘accelerated regulation’ was applied to a vaccine that was granted emergency use authorisation, expedited review designation, or orphan drug status at the time of its first approval, and ‘usual regulation’ otherwise. Data collection also included manufacturer origin type (small *vs*. large) and the number of incident cases attributable to VPDs. We defined large manufacturer origin as the top 20 biopharma companies based on their financial performance [26]. Due to data availability, we aggregated the number of incident cases attributable to VPDs by country, territory, and area (CTA) from the 2021 Global Burden of Disease study [27].

### Data analysis

We calculated the number of vaccines that received first-time regulatory approval globally between 2005 and 2024. To characterise the prophylactic vaccine development, we calculated the number of first-time approvals at the CTA level and stratified them by the approving country’s World Bank income group and WHO region. To assess the extent of non-availability, we calculated the total number of country-level vaccine approvals for the same period [15]. To evaluate delayed availability, we estimated approval delays as the time lag between global first approval and subsequent approvals in other countries [15]. We then constructed a linear regression model with robust standard errors to examine factors associated with approval delays. Due to small sample sizes, low-income countries (LICs) and lower-middle-income countries were combined into one group. Briefly, the outcome variable was defined as the number of days of approval delay per country during the analysis period. Covariates included manufacturer origin, income classification, number of incident cases attributable to VPDs, vaccine valence year of approval, and regulatory designation. Continuous covariates were log-transformed because of their strongly right-skewed distributions. The main analysis was performed on all vaccines, whereas the sub-analysis was performed on non-COVID-19 vaccines. *P*-values ≤0.05 were considered statistically significant. We conducted all analyses using *R*, version 4.3.2 (R Core Team, Vienna, Austria). Data manipulation was performed using the ‘tidyverse’ package. Multivariable linear regression was performed using base *R* functions, with robust standard errors estimated using the ‘sandwich’ and ‘lmtest’ packages. Visualisations were generated using ‘ggplot2’ package.

## RESULTS

### Vaccine approval

**Figure 1:**
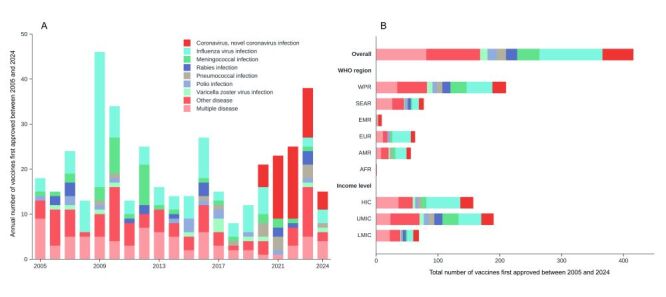
Number of vaccines first approved by indication between 2005 and 2024. **Panel A.** Annual number of vaccines first approved between 2005 and 2024 globally. **Panel B.** Total number of vaccines first approved between 2010 and 2024 by World Bank income group and WHO geographic region, respectively. AFR – African region, AMR – region of the Americas, EMR – Eastern Mediterranean region, EUR – European region, HIC – high-income country, LIC – low-income country, LMIC – lower-middle-income country, SEAR – South-East Asia region, UMIC – upper-middle-income country, WPR – Western Pacific region.

Between 2005 and 2024, 416 new prophylactic vaccines with regulatory approval globally met our inclusion criteria (**Figure 1**, Panel A, **Table 1**). We included a total of 2269 new prophylactic vaccine approvals or launches in our analysis across 123 CTAs, of which the dates for 153 approvals (6.7%) were approximated using the vaccine’s regulatory launch information due to a lack of exact approval dates.

**Table 1:** Characteristics of prophylactic vaccines approved between 2005 and 2024

Characteristics	n (%)
Total number of prophylactic vaccines approved	416 (100.0)
Vaccine type	
*Whole-pathogen*	123 (29.6)
*Split virus*	52 (12.5)
*Subunit*	188 (45.2)
*Nucleic acid*	24 (5.8)
*Multiple technology*	29 (7.0)
Valence	
*Monovalent*	203 (48.8)
*Multivalent and multipathogen*	213 (51.2)
Delivery route	
*Injectable*	385 (92.5)
*Novel delivery (oral, inhaled, or transdermal)*	31 (7.5)
Year of first approval	
*2005–2014*	218 (52.4)
*2015–2024*	198 (47.6)
Indications	
*Influenza virus infection*	102 (24.5)
*Multiple diseases*	81 (19.5)
*Coronavirus, novel coronavirus infection*	50 (12.0)
*Meningococcal infection*	36 (8.7)
*Rabies infection*	18 (4.3)
*Pneumococcal infection*	15 (3.6)
*Polio infection*	15 (3.6)
*Varicella zoster virus infection*	12 (2.9)
*Haemophilus influenzae infection*	9 (2.2)
*Japanese encephalitis virus infection*	9 (2.2)
*Typhoid infection*	8 (1.9)
*Hepatitis B virus infection*	7 (1.7)
*Human papillomavirus infection*	6 (1.4)
*Rotavirus infection*	6 (1.4)
*Tetanus infection*	6 (1.4)
*Ebola virus infection*	4 (1.0)
*Enterovirus 71 infection*	3 (0.7)
*Haemorrhagic fever infection*	3 (0.7)
*Hepatitis A virus infection*	3 (0.7)
*Measles infection*	3 (0.7)
*Cholera infection*	2 (0.5)
*Dengue virus infection*	2 (0.5)
*Malaria infection*	2 (0.5)
*Pertussis infection*	2 (0.5)
*Respiratory syncytial virus infection*	2 (0.5)
*Adenovirus, infection*	1 (0.2)
*Anthrax infection*	1 (0.2)
*Brucella infection*	1 (0.2)
*Chikungunya virus infection*	1 (0.2)
*Helicobacter pylori infection*	1 (0.2)
*Hepatitis E virus infection*	1 (0.2)
*Mumps infection*	1 (0.2)
*Rubella infection*	1 (0.2)
*Shigella infection*	1 (0.2)
*Tuberculosis infection*	1 (0.2)
Regulation status of the first approval	
*Usual regulation*	345 (82.9)
Accelerated regulation*	71 (17.1)
Number of CTAs in which a drug was approved in	
*1*	282 (67.8)
*2–5*	81 (19.5)
*6–10*	9 (2.2)
*>10*	44 (10.6)

CTA – countries, territories and areas

*Accelerated regulation means the prophylactic vaccine was granted emergency use authorisation, expedited review designation, or orphan drug status at the time of its first approval.

Across all vaccines, 188 (45.2%) used subunit technology, 123 (29.6%) used whole-pathogen technology, 52 (12.5%) used split-virus technology, 24 (5.8%) used nucleic acid technology, and 29 (7%) used multiple platforms. Most vaccines were multivalent and multipathogen (51.2%), received their first approval between 2005 and 2014 (52.4%), and were approved under usual regulatory pathways (82.9%). Regarding geographic spread, nearly two-thirds (67.8%) of vaccines were authorised in only one CTA, almost three times the proportion authorised in two to five CTAs and five times the proportion authorised in more than five CTAs.

Across all vaccines, the Western Pacific region accounted for the highest number of first approvals (n = 210), whereas the African region recorded only one (**Figure 1**, Panel B, **Table 2**). By World Bank income group, upper-middle-income countries (UMICs) led with 190 first approvals, followed by HICs (n = 157), lower-middle-income countries (n = 69), and none in LICs. At the country level, mainland China had the highest number of first approvals, followed by India and the USA (Table S1 in the **Online Supplementary Document**). Between 2005 and 2024, the European region recorded the highest total number of prophylactic vaccine approvals (**Table 2**), while the African region had the fewest. By World Bank income level, the total number of approvals ranged from just nine in LICs to 2668 in HICs. At the country level, mainland China led with 167 total approvals, followed by Hungary and Germany. The first and total approval patterns without COVID-19 vaccines yielded similar results (Table S2 in the **Online Supplementary Document**).

**Table 2:** Approval number and delay for prophylactic vaccines by country’s WHO region and World Bank Income group classification

Region	Approval number		Approval delay in days				
	**First, n**	**Total, n**	**MD (IQR)**	**n**	**≤180, n (%)**	**180–360, n (%)**	**>360, n (%)**
Overall	416	3312	213 (93, 768)	1853	845 (45.6)	331 (17.9)	677 (36.5)
WHO region							
*AFR*	1	56	307 (155, 613)	55	19 (34.5)	14 (25.5)	22 (40)
*AMR*	56	205	241 (102, 685)	149	63 (42.3)	34 (22.8)	52 (34.9)
*EMR*	9	64	193 (93, 1697)	55	27 (49.1)	9 (16.4)	19 (34.5)
*EUR*	63	2410	161 (84, 646)	1304	662 (50.8)	225 (17.3)	417 (32)
SE*AR*	77	143	291 (178, 1577)	66	18 (27.3)	19 (28.8)	29 (43.9)
*WPR*	210	434	705 (183, 2220)	224	56 (25)	30 (13.4)	138 (61.6)
Income level*							
*HIC*	157	2668	213 (85, 681)	1503	710 (47.2)	252 (16.8)	541 (36)
*UMIC*	190	437	268 (132, 1155)	212	81 (38.2)	50 (23.6)	81 (38.2)
*LMIC*	69	198	243 (132, 807)	129	52 (40.3)	28 (21.7)	49 (38)
*LIC*	0	9	471 (216, 713)	9	2 (22.2)	1 (11.1)	6 (66.7)

AFR – African region, AMR – region of the Americas, EMR – Eastern Mediterranean region, EUR – European region, HIC – high-income country, LIC – low-income country, LMIC – lower-middle-income country, SEAR – South-East Asia region, UMIC – upper-middle-income country, WPR – Western Pacific region, MD – median, IQR – interquartile range

*Income level source: World Bank (2024).

### Vaccine approval delays

**Figure 2:**
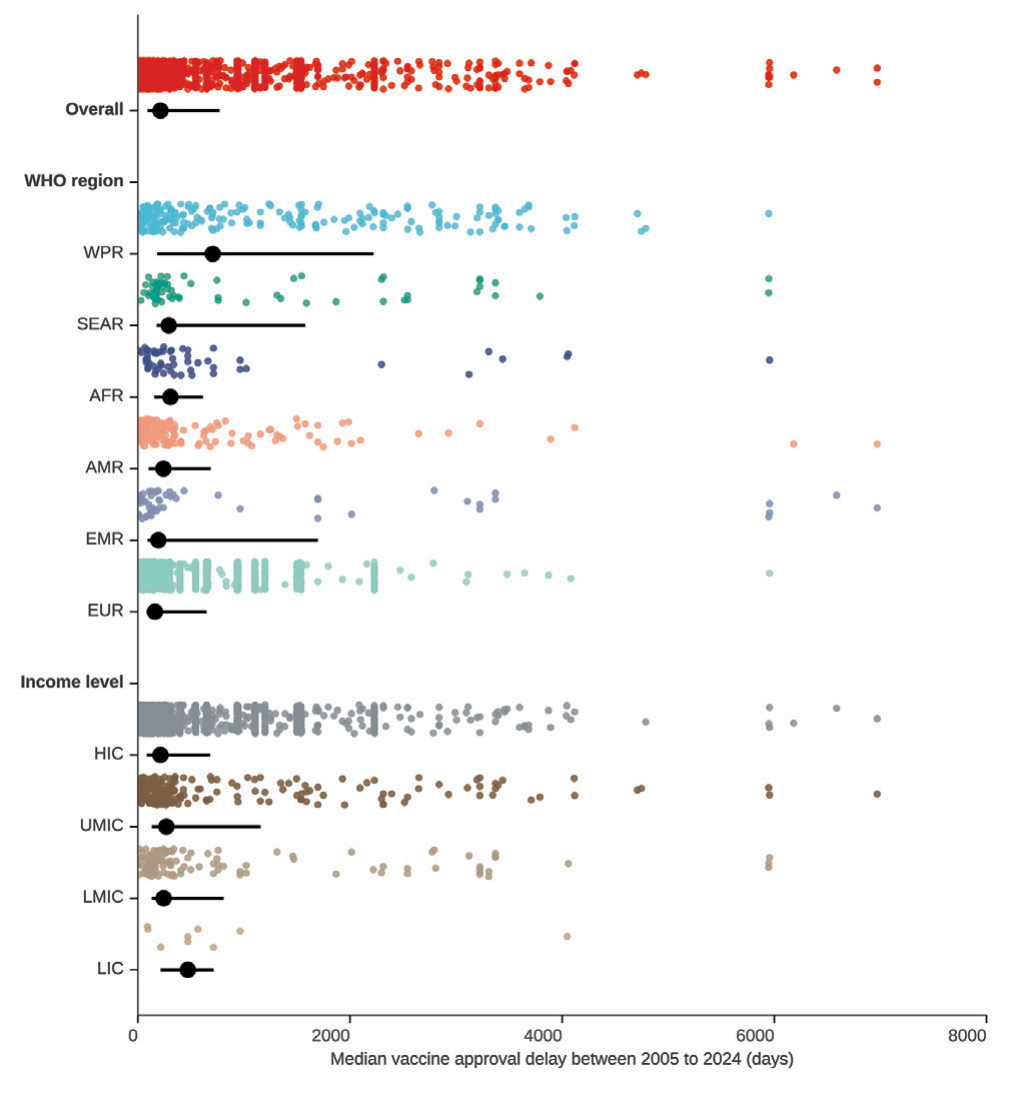
Median of vaccine approval delay (days) between 2005 and 2024, by country’s WHO region and World Bank income group. Each coloured dot represents one country, territory, or area. The large black dot represents the median, and the lines represent the interquartile range. AFR – African region, AMR – region of the Americas, EMR – Eastern Mediterranean region, EUR – European region, HIC – high-income country, LIC – low-income country, LMIC – lower-middle-income country, SEAR – South-East Asia region, UMIC – upper-middle-income country, WPR – Western Pacific region.

Globally, the MD approval delay for all prophylactic vaccines was 213.0 days (interquartile range (IQR) = 93.0, 768.0), with substantial variation across WHO regions – from 705.5 days (IQR = 183.3, 2220.3) in the Western Pacific to 161.0 days (IQR = 84.0, 646.0) in the European region. By income group, MD delays were 471.0 days (IQR = 216.0, 713.0) in LICs, nearly twice that in HICs (**Figure 2**, **Table 2**). At the country level, the longest MD delays were observed in Mongolia, Brunei, and Saudi Arabia. However, excluding COVID-19 vaccines, these processes took much longer: specifically, the MD time from initial approval to subsequent approvals was 299 days (Table S1–3 in the **Online Supplementary Document**).

By specific disease, the most common single-disease targets were influenza virus infection (24%), novel coronavirus infection (12%), and meningococcal infection (8%). Additionally, 19% targeted multiple indications (**Table 1**). The highest MD approval delays in days were observed for vaccines against cholera (MD = 3004.0 days; IQR = 2232.5, 3055.5), enterovirus 71 (MD = 2825.0 days; IQR = 2669.5, 2980.5), and hepatitis E (MD = 2015.0 days). In contrast, vaccines targeting the Ebola virus, respiratory syncytial virus, novel coronavirus, and Japanese encephalitis virus had MD delays <100 days (Table S4 in the **Online Supplementary Document**).

Our exploratory multivariable linear regression with robust standard errors suggested that income classification, vaccine valence, year of first approval, and regulatory designation may be associated with approval delays (**Table 3**). Compared with HICs, approval delays were approximately 377 days longer in UMICs and 479 days longer in low- and lower-middle-income countries. Vaccines first approved during 2015–2024 showed shorter delays compared with those first approved during 2005–2014. Regarding vaccine valence, multivalent and multipathogen vaccines were associated with longer approval delays than monovalent vaccines. In terms of regulatory pathway, vaccines granted accelerated designation had shorter approval delays than those following the usual regulatory process. Regression models without COVID-19 products yielded broadly similar results; however, vaccines originating from large manufacturers were associated with shorter approval delays than those from small manufacturers. Regression models with exact approval dates yielded similar results (Table S5 and S6 in the **Online Supplementary Document**).

**Table 3:** Multivariable linear regression analysis of approval delays with robust standard errors

Variable	Adjusted difference (95% CI)	*P*-value
Income level		
*HIC*	Ref.	
*UMIC*	376.71 (219.53, 533.25)	<0.0001
*LMIC and LIC*	478.80 (269.23, 687.37)	<0.0001
Vaccine valence		
*Monovalent vaccines*	Ref.	
*Multivalent and multipathogen vaccines*	152.58 (57.53, 247.84)	0.002
Regulation status of the first approval		
*Usual regulation*	Ref.	
*Accelerated regulation*	–364.93 (–456.45, –273.40)	<0.0001
Year of first approval		
*2005–2014*	Ref.	
*2015–2024*	–262.19 (–363.04, –161.34)	<0.0001
Manufacturer origin type		
*Small*	Ref.	
*Large-size*	73.04 (–15.35, 161.44)	0.105
Log_2_ (VPDs incidence cases)	12.71 (–4.07, 29.51)	0.138

CI – confidence interval, ref – reference, VPDs – vaccine-preventable diseases

## DISCUSSION

This study presents a comprehensive analysis of regulatory approvals for commercially developed prophylactic vaccines over the past two decades. Our analysis reveals substantial disparities in both the volume and delays of vaccine approvals across regions and income levels. Countries with fewer resources – particularly in Africa and parts of the Western Pacific – experienced markedly longer approval delays compared with high-income settings. These patterns suggest that regulatory approval pathways may represent an important upstream contributor to global inequities in vaccine availability.

These findings should be interpreted within the broader pathway linking vaccine development to population-level access. Regulatory approval represents a necessary but not sufficient condition for equitable vaccine availability. Even after authorisation, access may remain constrained by financing limitations, procurement policies, market incentives, and distribution capacity [7].

We found that vaccines approved in low- and lower-middle-income countries experienced longer delays than those in HICs, which aligns with other qualitative evidence [15]. The regional patterns observed in this study likely reflect differences in regulatory capacity, market size, and industry engagement across countries [5]. High-income countries often have well-resourced regulatory authorities and represent major pharmaceutical markets, creating incentives for manufacturers to prioritise regulatory submissions [28]. In contrast, regulatory agencies in many LMICs face substantial resource constraints, including limited technical staff, funding shortages, and fragmented regulatory procedures [29,30]. These structural factors may contribute to longer approval timelines and fewer vaccine approvals in resource-limited settings.

We observed a reduction in the median approval delays for prophylactic vaccines approved between 2015 and 2024, compared to those approved in the preceding decade. This pattern may reflect several global developments, including increasing reliance on regulatory practices, greater international collaboration among regulatory authorities, and the growing adoption of accelerated approval pathways [31,32]. While our study does not directly evaluate the impact of these mechanisms, the observed temporal trend suggests that regulatory systems may be gradually becoming more responsive to the need for timely access to vaccines.

Differences in approval timelines across vaccine types were also observed. Multipathogen and multivalent vaccines experienced longer approval delays than monovalent vaccines. In certain local NRAs, conservative regulatory requirements and potential conflicts with existing immunisation schedules may present significant challenges to the approval of combination vaccines [25]. This underscores the need for targeted, evidence-based regulatory strategies to expedite the approval of multipathogen and multivalent vaccines, particularly in regions with structural regulatory barriers [25].

Although our study does not directly assess specific regulatory harmonisation initiatives, the disparities identified here suggest that strengthening regulatory capacity and facilitating reliance on decisions from well-functioning regulatory authorities may represent promising approaches to reducing approval delays in resource-limited settings. Regional collaboration, information sharing, and technical assistance programmes may also help improve the efficiency of regulatory review processes [10,33].

The findings may also have implications for global pandemic preparedness. Our results showed that vaccines approved through standard procedures experienced longer delays than those granted accelerated approvals – a pattern likely influenced by the global regulatory response during the COVID-19 pandemic [34]. In pandemic preparedness settings, investments in strengthening effective regulatory functions and systems, aligned with the Global Benchmarking Tool, may offer a promising mechanism for accelerating global regulatory approvals [6,10,32]. Beyond pandemic preparedness, our disease-specific analysis identified notably long approval delays for vaccines targeting certain neglected diseases predominantly affecting LMICs, suggesting the potential need for differentiated regulatory attention for non-pandemic vaccine-preventable diseases.

This study has several limitations. First, this study equates vaccine availability with regulatory approval, which does not capture whether a vaccine was subsequently procured, introduced into national immunisation programmes, or made widely accessible to target populations. Our findings, therefore, reflect the regulatory dimension of vaccine availability rather than the full spectrum of access [15]. Second, the data set focused on commercially developed prophylactic vaccines, and the exclusion of publicly developed vaccines may introduce uncertainty about the full global distribution of regulatory approvals. Third, the classification of new prophylactic vaccines in Pharmaprojects includes vaccines with updated formulations or strain modifications; future studies may consider restricting analyses to vaccines with novel antigens to enable more precise comparisons. Finally, approval information relied on voluntary reporting by manufacturers or public sources; we supplemented missing approval data with launch data where possible [15] and cross-checked with additional sources, but both remain imperfect proxies for actual availability.

## CONCLUSIONS

In conclusion, substantial disparities persist in the regulatory approval of prophylactic vaccines across regions and income groups. Countries with resource-limited settings – particularly in Africa and the Western Pacific – experienced the greatest inequities. While regulatory approval is only one step in the pathway to vaccine access, reducing approval delays may be an important upstream opportunity to improve the equity of global vaccine availability.

## Additional material


Online Supplementary Document
