# Supplementary material for: Global disparities in regulatory approval of prophylactic vaccines, 2005–2024
Source: J Glob Health. 2026 Jul 10;16:04145. doi: 10.7189/jogh.16.04145 (PMC13351647; doi:10.7189/jogh.16.04145)
Supplement: Online Supplementary Document [file jogh-16-04145-s001.pdf]

Supplement to: Wang R, Chen J, Huang Y, Xu M. Global disparities in regulatory approval of prophylactic vaccines, 2005–2024. J Glob Health. 2026;16:04145.

Supplementary appendix

Appendix 1. Supplementary tables and figures 2
Table S1 Approval number and delay of new prophylactic vaccines by countries, territories and areas 2
Table S2 Approval number and delay for prophylactic vaccines (without COVID-19 vaccines) by WHO region and World Bank Income group classification..................6
Table S3 Approval number and delay of new prophylactic vaccines (without COVID-19 vaccines) by countries, territories and areas...7
Table S4 Approval delay of new prophylactic vaccines by indications globally 10
Table S5: Multivariable negative binomial regression analysis on approval delays estimated by linear regressions with robust standard errors (without COVID-19 vaccines)..11
Table S6: Multivariable negative binomial regression analysis on approval delays estimated by linear regressions with robust standard errors (using original data). ..12
Appendix 2. The STROBE reporting checklist 13

Appendix 1. Supplementary tables and figures

Table S1 Approval number and delay of new prophylactic vaccines by countries, territories and areas

Country	World Bank income group[a]	WHO region	Approval number		Approval delay in days			
			First[b] (N)	Total (N)	Overall [Median (Interquartile range); N]	≤180 days [n (%)]	180-360 days [n (%)]	>360 days [n (%)]
Albania	EUR	UMIC	0	1	39 (NA); 1	1 (100)	0	0
Argentina	AMR	UMIC	0	9	348 (1211); 9	2 (22.2)	3 (33.3)	4 (44.4)
Australia	WPR	HIC	7	45	454.5 (1381.3); 38	8 (21.1)	10 (26.3)	20 (52.6)
Austria	EUR	HIC	36	77	161 (562); 41	21 (51.2)	7 (17.1)	13 (31.7)
Bahrain	EMR	HIC	2	6	180.5 (1683.8); 4	2 (50)	1 (25)	1 (25)
Bangladesh	SEAR	LMIC	0	7	226 (355); 7	2 (28.6)	3 (42.9)	2 (28.6)
Belarus	EUR	UMIC	1	2	341 (NA); 1	0	1 (100)	0
Belgium	EUR	HIC	36	79	189 (569.5); 43	21 (48.8)	7 (16.3)	15 (34.9)
Bolivia	AMR	LMIC	0	1	300 (NA); 1	0	1 (100)	0
Botswana	AFR	UMIC	0	3	107 (36); 3	3 (100)	0	0
Brazil	AMR	UMIC	0	14	219.5 (1511.3); 14	7 (50)	2 (14.3)	5 (35.7)
Brunei	WPR	HIC	0	3	3224 (1611); 3	1 (33.3)	0	2 (66.7)
Bulgaria	EUR	UMIC	35	75	157 (490); 40	21 (52.5)	7 (17.5)	12 (30)
Burkina Faso	AFR	LIC	0	1	216 (NA); 1	0	1 (100)	0
Cambodia	WPR	LMIC	0	1	300 (NA); 1	0	1 (100)	0
Cameroon	AFR	LMIC	0	2	2010.5 (1297.5); 2	0	0	2 (100)
Canada	AMR	HIC	3	36	312 (732); 33	11 (33.3)	8 (24.2)	14 (42.4)
Chad	AFR	LIC	0	1	713 (NA); 1	0	0	1 (100)
Chile	AMR	HIC	1	10	405 (612); 9	3 (33.3)	1 (11.1)	5 (55.6)
Mainland China	WPR	UMIC	156	167	1236 (2894); 11	2 (18.2)	0	9 (81.8)
Colombia	AMR	UMIC	0	7	358 (376); 7	2 (28.6)	2 (28.6)	3 (42.9)
Costa Rica	AMR	UMIC	0	2	104 (91); 2	1 (50)	1 (50)	0
Croatia	EUR	HIC	35	75	157 (490); 40	21 (52.5)	7 (17.5)	12 (30)
Cuba	AMR	UMIC	4	5	68 (NA); 1	1 (100)	0	0
Cyprus	EUR	HIC	35	75	157 (490); 40	21 (52.5)	7 (17.5)	12 (30)
Czechia	EUR	HIC	35	75	157 (490); 40	21 (52.5)	7 (17.5)	12 (30)
Democratic Republic of the Congo	AFR	LIC	0	2	2071 (1976); 2	1 (50)	0	1 (50)
Denmark	EUR	HIC	36	77	161 (562); 41	21 (51.2)	7 (17.1)	13 (31.7)
Dominican Republic	AMR	UMIC	0	3	163 (416); 3	2 (66.7)	0	1 (33.3)

Ecuador	AMR	UMIC	0	3	181 (169); 3	1 (33.3)	2 (66.7)	0
Egypt	EMR	LMIC	0	4	301.5 (760.3); 4	1 (25)	1 (25)	2 (50)
El Salvador	AMR	UMIC	0	1	62 (NA); 1	1 (100)	0	0
Estonia	EUR	HIC	35	75	175 (562); 40	20 (50)	7 (17.5)	13 (32.5)
Eswatini	AFR	LMIC	0	1	88 (NA); 1	1 (100)	0	0
Finland	EUR	HIC	35	76	161 (562); 41	21 (51.2)	7 (17.1)	13 (31.7)
France	EUR	HIC	36	77	161 (562); 41	21 (51.2)	7 (17.1)	13 (31.7)
Germany	EUR	HIC	36	80	175 (562); 44	22 (50)	7 (15.9)	15 (34.1)
Ghana	AFR	LMIC	1	4	219 (202); 3	1 (33.3)	1 (33.3)	1 (33.3)
Greece	EUR	HIC	35	77	175 (536.5); 42	21 (50)	7 (16.7)	14 (33.3)
Guatemala	AMR	UMIC	0	3	278 (114); 3	1 (33.3)	2 (66.7)	0
Guinea	AFR	LMIC	0	1	471 (NA); 1	0	0	1 (100)
Guyana	AMR	HIC	0	3	303 (231); 3	1 (33.3)	1 (33.3)	1 (33.3)
Hong Kong, China	WPR	HIC	0	15	655 (1846.5); 15	3 (20)	2 (13.3)	10 (66.7)
Hungary	EUR	HIC	37	80	154 (386.5); 43	23 (53.5)	8 (18.6)	12 (27.9)
Iceland	EUR	HIC	35	74	161 (511); 39	20 (51.3)	7 (17.9)	12 (30.8)
India	SEAR	LMIC	63	72	1021 (1282); 9	2 (22.2)	2 (22.2)	5 (55.6)
Indonesia	SEAR	UMIC	12	26	252.5 (2263.5); 14	2 (14.3)	6 (42.9)	6 (42.9)
Iran	EMR	LMIC	3	8	203 (3048); 5	2 (40)	1 (20)	2 (40)
Iraq	EMR	UMIC	0	3	126 (1541); 3	2 (66.7)	0	1 (33.3)
Ireland	EUR	HIC	35	77	175 (564); 42	21 (50)	7 (16.7)	14 (33.3)
Israel	EUR	HIC	0	6	1203 (3043.8); 6	2 (33.3)	1 (16.7)	3 (50)
Italy	EUR	HIC	35	76	161 (562); 41	21 (51.2)	7 (17.1)	13 (31.7)
Japan	WPR	HIC	20	46	589 (1622.3); 26	11 (42.3)	1 (3.8)	14 (53.8)
Jordan	EMR	LMIC	0	3	42 (51.5); 3	3 (100)	0	0
Kazakhstan	EUR	UMIC	0	1	264 (NA); 1	0	1 (100)	0
Kenya	AFR	LMIC	0	8	499.5 (1305); 8	1 (12.5)	3 (37.5)	4 (50)
Kuwait	EMR	HIC	0	3	103 (843); 3	2 (66.7)	0	1 (33.3)
Kyrgyzstan	EUR	LMIC	0	1	435 (NA); 1	0	0	1 (100)
Laos	WPR	LMIC	0	1	149 (NA); 1	1 (100)	0	0
Latvia	EUR	HIC	35	75	157 (490); 40	21 (52.5)	7 (17.5)	12 (30)
Lebanon	EMR	LMIC	0	1	167 (NA); 1	1 (100)	0	0
Libya	EMR	UMIC	0	1	69 (NA); 1	1 (100)	0	0
Liechtenstein	EUR	HIC	35	74	161 (511); 39	20 (51.3)	7 (17.9)	12 (30.8)
Lithuania	EUR	HIC	35	75	157 (490); 40	21 (52.5)	7 (17.5)	12 (30)
Luxembourg	EUR	HIC	35	76	161 (561); 41	21 (51.2)	7 (17.1)	13 (31.7)
Macao, China	WPR	HIC	0	4	941.5 (1660.5); 4	1 (25)	1 (25)	2 (50)

Malaysia	WPR	UMIC	0	19	1544 (2414.5); 19	1 (5.3)	5 (26.3)	13 (68.4)
Maldives	SEAR	UMIC	0	4	133.5 (67.3); 4	3 (75)	1 (25)	0
Mali	AFR	LIC	0	1	566 (NA); 1	0	0	1 (100)
Malta	EUR	HIC	35	75	157 (490); 40	21 (52.5)	7 (17.5)	12 (30)
Mauritius	AFR	UMIC	0	1	77 (NA); 1	1 (100)	0	0
Mexico	AMR	UMIC	2	16	232 (807.3); 14	6 (42.9)	3 (21.4)	5 (35.7)
Mongolia	WPR	LMIC	1	4	3309 (73.5); 3	0	0	3 (100)
Morocco	EMR	LMIC	0	4	140.5 (255); 4	3 (75)	0	1 (25)
Myanmar	SEAR	LMIC	0	4	2883 (1229.3); 4	0	0	4 (100)
Nepal	SEAR	LMIC	0	4	218 (263.5); 4	2 (50)	1 (25)	1 (25)
Netherlands	EUR	HIC	35	76	161 (562); 41	21 (51.2)	7 (17.1)	13 (31.7)
New Zealand	WPR	HIC	0	18	1101.5 (1885.5); 18	5 (27.8)	0	13 (72.2)
Nicaragua	AMR	LMIC	0	3	107 (12.5); 3	3 (100)	0	0
Nigeria	AFR	LMIC	0	7	204 (413); 7	3 (42.9)	2 (28.6)	2 (28.6)
Norway	EUR	HIC	35	75	175 (562); 40	20 (50)	7 (17.5)	13 (32.5)
Oman	EMR	HIC	0	3	300 (3383); 3	0	2 (66.7)	1 (33.3)
Pakistan	EMR	LMIC	0	7	757 (2414); 7	2 (28.6)	1 (14.3)	4 (57.1)
Panama	AMR	HIC	0	2	118.5 (105.5); 2	1 (50)	1 (50)	0
Paraguay	AMR	UMIC	0	5	206 (75); 5	2 (40)	3 (60)	0
Peru	AMR	UMIC	0	6	143.5 (136.3); 6	4 (66.7)	1 (16.7)	1 (16.7)
Philippines	WPR	LMIC	0	16	221.5 (1556.8); 16	8 (50)	1 (6.2)	7 (43.8)
Poland	EUR	HIC	35	77	175 (564); 42	21 (50)	7 (16.7)	14 (33.3)
Portugal	EUR	HIC	35	76	161 (562); 41	21 (51.2)	7 (17.1)	13 (31.7)
Qatar	EMR	HIC	0	3	50 (839); 3	2 (66.7)	0	1 (33.3)
Republic of Moldova	EUR	UMIC	0	1	247 (NA); 1	0	1 (100)	0
Romania	EUR	HIC	35	75	157 (490); 40	21 (52.5)	7 (17.5)	12 (30)
Russia Federation	EUR	UMIC	13	18	1155 (1221); 5	0	0	5 (100)
Rwanda	AFR	LIC	0	3	471 (436.5); 3	1 (33.3)	0	2 (66.7)
Saint Vincent and the Grenedines	AMR	UMIC	0	1	172 (NA); 1	1 (100)	0	0
Saudi Arabia	EMR	HIC	1	5	3077 (5960.8); 4	1 (25)	1 (25)	2 (50)
Serbia	EUR	UMIC	0	2	73.5 (51.5); 2	2 (100)	0	0
Seychelles	AFR	HIC	0	1	163 (NA); 1	1 (100)	0	0
Singapore	WPR	HIC	0	24	1045.5 (1846.3); 24	4 (16.7)	1 (4.2)	19 (79.2)
Slovakia	EUR	HIC	35	75	157 (485.5); 40	21 (52.5)	7 (17.5)	12 (30)
Slovenia	EUR	HIC	35	75	157 (485.5); 40	21 (52.5)	7 (17.5)	12 (30)
South Africa	AFR	UMIC	0	8	312.5 (702.8); 8	2 (25)	3 (37.5)	3 (37.5)

South Korea	WPR	HIC	22	44	723 (1613); 22	5 (22.7)	2 (9.1)	15 (68.2)
Spain	EUR	HIC	36	78	175 (564); 42	21 (50)	7 (16.7)	14 (33.3)
Sri Lanka	SEAR	LMIC	0	3	235 (83.5); 3	1 (33.3)	2 (66.7)	0
Sweden	EUR	HIC	35	76	189 (561); 41	20 (48.8)	7 (17.1)	14 (34.1)
Switzerland	EUR	HIC	0	15	536 (1442); 15	7 (46.7)	0	8 (53.3)
Taiwan, China	WPR	HIC	4	19	1155 (2338.5); 15	3 (20)	3 (20)	9 (60)
Thailand	SEAR	UMIC	2	23	380 (1411); 21	6 (28.6)	4 (19)	11 (52.4)
Tunisia	EMR	LMIC	0	3	83 (67); 3	3 (100)	0	0
Turkey	EUR	UMIC	0	6	246.5 (1122.8); 6	3 (50)	1 (16.7)	2 (33.3)
Turkmenistan	EUR	UMIC	0	1	294 (NA); 1	0	1 (100)	0
UK	EUR	HIC	33	69	201 (467.8); 36	17 (47.2)	7 (19.4)	12 (33.3)
USA	AMR	HIC	46	70	259 (1030.3); 24	10 (41.7)	3 (12.5)	11 (45.8)
Uganda	AFR	LIC	0	1	471 (NA); 1	0	0	1 (100)
Ukraine	EUR	LMIC	0	3	130 (55.5); 3	2 (66.7)	1 (33.3)	0
United Arab Emirates	EMR	HIC	3	10	334 (906); 7	2 (28.6)	2 (28.6)	3 (42.9)
United Republic of Tanzania	AFR	LMIC	0	5	328 (99); 5	0	3 (60)	2 (40)
Uzbekistan	EUR	LMIC	1	1	NA	NA	NA	NA
Venezuela	AMR	LMIC	0	5	167 (257); 5	3 (60)	0	2 (40)
Vietnam	WPR	LMIC	0	8	227 (799.5); 8	3 (37.5)	3 (37.5)	2 (25)
Zambia	AFR	LMIC	0	1	95 (NA); 1	1 (100)	0	0
Zimbabwe	AFR	LMIC	0	5	175 (40); 5	3 (60)	1 (20)	1 (20)

a Source: World Bank (2024);
b One vaccine could receive its first global approval in multiple countries when the regulatory approval agency is the European Commission.
Abbreviation: AFR, African region. AMR, region of the Americas. EMR, Eastern Mediterranean region. EUR, European region. SEAR, South-East Asia region. WPR, Western Pacific region. HIC, High-income country. LIC, low-income country. LMIC, lower-middle-income country. UMIC, upper-middle-income country. NA, not available.

Table S2 Approval number and delay for prophylactic vaccines (without COVID-19 vaccines) by WHO region and World Bank Income group classification

	Approval number		Approval delay in days			
	First (N)	Total (N)	Overall [Median (Interquartile range); N]	≤180 days [n (%)]	180-360 days [n (%)]	>360 days [n (%)]
Overall	364	2615	299(956);1324	442(33.4)	257(19.4)	625(47.2)
WHO region						
AFR	1	24	713(2332.5);23	4(17.4)	2(8.7)	17(73.9)
AMR	49	130	569(1134);81	18(22.2)	16(19.8)	47(58)
EMR	2	21	3107(2962.5);19	2(10.5)	0	17(89.5)
EUR	55	1988	251(791);1006	405(40.3)	217(21.6)	384(38.2)
SEAR	69	102	1589(2452);33	2(6.1)	4(12.1)	27(81.8)
WPR	188	350	1532(2143.5);162	11(6.8)	18(11.1)	133(82.1)
Income level[a]						
HIC	135	2180	273(885);1149	412(35.9)	234(20.4)	503(43.8)
UMIC	170	318	938(2046);117	23(19.7)	19(16.2)	75(64.1)
LMIC	59	109	1669.5(2503);50	6(12)	3(6)	41(82)
LIC	0	8	518.5(368.5);8	1(12.5)	1(12.5)	6(75)

a Income level source: World Bank (2024);

Abbreviation: AFR, African region; AMR, region of the Americas; EMR, Eastern Mediterranean region; EUR, European region; SEAR, South-East Asia region; WPR, Western Pacific region; HIC, High-income country; LIC, low-income country; LMIC, lower-middle-income country; UMIC, upper-middle-income country.

Table S3 Approval number and delay of new prophylactic vaccines (without COVID-19 vaccines) by countries, territories and areas

Country	World Bank income group[a]	WHO region	Approval number		Approval delay in days			
			First[b] (N)	Total (N)	Overall [Median (Interquartile range); N]	≤180 days [n (%)]	180-360 days [n (%)]	>360 days [n (%)]
Argentina	AMR	UMIC	0	5	1496(382)5	0	1(20)	4(80)
Australia	WPR	HIC	6	37	708(1428.5)31	2(6.5)	9(29)	20(64.5)
Austria	EUR	HIC	32	64	242.5(797.8)32	13(40.6)	7(21.9)	12(37.5)
Bahrain	EMR	HIC	0	1	5948(NA)1	0	0	1(100)
Bangladesh	SEAR	LMIC	0	2	1527(770)2	0	0	2(100)
Belarus	EUR	UMIC	1	1				
Belgium	EUR	HIC	32	66	251.5(745.5)34	13(38.2)	7(20.6)	14(41.2)
Bolivia	AMR	LMIC	0	1	300(NA)1	0	1(100)	0
Brazil	AMR	UMIC	0	10	919(1514.8)10	4(40)	1(10)	5(50)
Brunei	WPR	HIC	0	2	3297.5(73.5)2	0	0	2(100)
Bulgaria	EUR	UMIC	0	62	234(655.5)31	13(41.9)	7(22.6)	11(35.5)
Burkina Faso	AFR	LIC	0	1	216(NA)1	0	1(100)	0
Cambodia	WPR	LMIC	0	1	300(NA)1	0	1(100)	0
Cameroon	AFR	LMIC	0	2	2010.5(1297.5)2	0	0	2(100)
Canada	AMR	HIC	2	27	408(845)25	4(16)	8(32)	13(52)
Chad	AFR	LIC	0	1	713(NA)1	0	0	1(100)
Chile	AMR	HIC	1	6	757(1156)5	0	0	5(100)
China	WPR	UMIC	141	149	2647.5(2861.5)8	0	0	8(100)
Colombia	AMR	UMIC	0	2	587(158)2	0	0	2(100)
Costa Rica	AMR	UMIC	0	1	195(NA)1	0	1(100)	0
Croatia	EUR	HIC	31	62	234(655.5)31	13(41.9)	7(22.6)	11(35.5)
Cuba	AMR	UMIC	2	2				
Cyprus	EUR	HIC	31	62	234(655.5)31	13(41.9)	7(22.6)	11(35.5)
Czechia	EUR	HIC	31	62	234(655.5)31	13(41.9)	7(22.6)	11(35.5)
Democratic Republic of the Congo	AFR	LIC	0	2	2071(1976)2	1(50)	0	1(50)
Denmark	EUR	HIC	32	64	242.5(588.5)32	13(40.6)	7(21.9)	12(37.5)
Dominican Republic	AMR	UMIC	0	1	888(NA)1	0	0	1(100)
Egypt	EMR	LMIC	0	1	2794(NA)1	0	0	1(100)
El Salvador	AMR	UMIC	0	1	62(NA)1	1(100)	0	0
Estonia	EUR	HIC	31	62	234(655.5)31	13(41.9)	7(22.6)	11(35.5)

Finland	EUR	HIC	31	63	242.5(588.5)32	13(40.6)	7(21.9)	12(37.5)
France	EUR	HIC	32	64	242.5(797.8)32	13(40.6)	7(21.9)	12(37.5)
Germany	EUR	HIC	32	67	251(662)35	14(40)	7(20)	14(40)
Ghana	AFR	LMIC	1	2	95(NA)1	1(100)	0	0
Greece	EUR	HIC	31	64	251(688)33	13(39.4)	7(21.2)	13(39.4)
Guatemala	AMR	UMIC	0	1	278(NA)1	0	1(100)	0
Guinea	AFR	LMIC	0	1	471(NA)1	0	0	1(100)
Guyana	AMR	HIC	0	1	625(NA)1	0	0	1(100)
Hong Kong, China	WPR	HIC	0	13	1540(1884)13	1(7.7)	2(15.4)	10(76.9)
Hungary	EUR	HIC	33	64	234(655.5)31	13(41.9)	7(22.6)	11(35.5)
Iceland	EUR	HIC	31	62	234(655.5)31	13(41.9)	7(22.6)	11(35.5)
India	SEAR	LMIC	58	64	1391(675.5)6	0	1(16.7)	5(83.3)
Indonesia	SEAR	UMIC	9	15	2528(689.5)6	0	1(16.7)	5(83.3)
Iran	EMR	LMIC	0	2	3297.5(73.5)2	0	0	2(100)
Iraq	EMR	UMIC	0	1	3107(NA)1	0	0	1(100)
Ireland	EUR	HIC	31	64	251(791)33	13(39.4)	7(21.2)	13(39.4)
Israel	EUR	HIC	0	3	3481(779.5)3	0	0	3(100)
Italy	EUR	HIC	31	63	242.5(588.5)32	13(40.6)	7(21.9)	12(37.5)
Japan	WPR	HIC	17	36	885(1827.5)19	4(21.1)	1(5.3)	14(73.7)
Kenya	AFR	LMIC	0	3	3122(2466.5)3	0	0	3(100)
Kuwait	EMR	HIC	0	1	1697(NA)1	0	0	1(100)
Latvia	EUR	HIC	31	62	234(655.5)31	13(41.9)	7(22.6)	11(35.5)
Liechtenstein	EUR	HIC	31	62	234(655.5)31	13(41.9)	7(22.6)	11(35.5)
Lithuania	EUR	HIC	31	62	234(655.5)31	13(41.9)	7(22.6)	11(35.5)
Luxembourg	EUR	HIC	31	63	242.5(797.8)32	13(40.6)	7(21.9)	12(37.5)
Macao, China	WPR	HIC	0	3	1540(1396.5)3	0	1(33.3)	2(66.7)
Malaysia	WPR	UMIC	0	13	2393(1680)13	0	1(7.7)	12(92.3)
Mali	AFR	LIC	0	1	566(NA)1	0	0	1(100)
Malta	EUR	HIC	31	62	234(655.5)31	13(41.9)	7(22.6)	11(35.5)
Mexico	AMR	UMIC	2	8	1095.5(1799.8)6	1(16.7)	0	5(83.3)
Mongolia	WPR	LMIC	0	3	3309(73.5)3	0	0	3(100)
Morocco	EMR	LMIC	0	1	964(NA)1	0	0	1(100)
Myanmar	SEAR	LMIC	0	3	3224(414.5)3	0	0	3(100)
Nepal	SEAR	LMIC	0	1	757(NA)1	0	0	1(100)
Netherlands	EUR	HIC	31	63	242.5(588.5)32	13(40.6)	7(21.9)	12(37.5)
New Zealand	WPR	HIC	0	15	1524(1705)15	2(13.3)	0	13(86.7)
Nigeria	AFR	LMIC	0	3	713(480)3	1(33.3)	0	2(66.7)
Norway	EUR	HIC	31	63	242.5(588.5)32	13(40.6)	7(21.9)	12(37.5)
Oman	EMR	HIC	0	1	6970(NA)1	0	0	1(100)

Pakistan	EMR	LMIC	0	4	2619.5(1560.3)4	0	0	4(100)
Paraguay	AMR	UMIC	0	1	251(NA)1	0	1(100)	0
Peru	AMR	UMIC	0	2	341.5(63.5)2	0	1(50)	1(50)
Philippines	WPR	LMIC	0	8	1840(1579.5)8	1(12.5)	0	7(87.5)
Poland	EUR	HIC	31	64	251(791)33	13(39.4)	7(21.2)	13(39.4)
Portugal	EUR	HIC	31	63	242.5(588.5)32	13(40.6)	7(21.9)	12(37.5)
Qatar	EMR	HIC	0	1	1697(NA)1	0	0	1(100)
Romania	EUR	HIC	31	62	234(655.5)31	13(41.9)	7(22.6)	11(35.5)
Russia Federation	EUR	UMIC	13	18	1155(1221)5	0	0	5(100)
Rwanda	AFR	LIC	0	2	717.5(246.5)2	0	0	2(100)
Saudi Arabia	EMR	HIC	1	3	6271(316)2	0	0	2(100)
Singapore	WPR	HIC	0	19	1540(1855)19	0	1(5.3)	18(94.7)
Slovakia	EUR	HIC	31	62	234(655.5)31	13(41.9)	7(22.6)	11(35.5)
Slovenia	EUR	HIC	31	62	234(655.5)31	13(41.9)	7(22.6)	11(35.5)
South Africa	AFR	UMIC	0	3	2297(1578)3	0	1(33.3)	2(66.7)
South Korea	WPR	HIC	21	36	1434(2194)15	0	1(6.7)	14(93.3)
Spain	EUR	HIC	31	64	251(791)33	13(39.4)	7(21.2)	13(39.4)
Sweden	EUR	HIC	31	63	242.5(588.5)32	13(40.6)	7(21.9)	12(37.5)
Switzerland	EUR	HIC	0	10	1162.5(1193)10	2(20)	0	8(80)
Taiwan, China	WPR	HIC	3	13	2060.5(1393)10	1(10)	1(10)	8(80)
Thailand	SEAR	UMIC	2	17	1347(2396.5)15	2(13.3)	2(13.3)	11(73.3)
Tunisia	EMR	LMIC	0	2	129.5(46.5)2	2(100)	0	0
Turkey	EUR	UMIC	0	5	317(1413)5	2(40)	1(20)	2(40)
UK	EUR	HIC	31	59	286(680)28	10(35.7)	6(21.4)	12(42.9)
USA	AMR	HIC	42	60	478(1383.3)18	8(44.4)	1(5.6)	9(50)
Uganda	AFR	LIC	0	1	471(NA)1	0	0	1(100)
United Arab Emirates	EMR	HIC	1	3	3826(2129)2	0	0	2(100)
Venezuela	AMR	LMIC	0	1	404(NA)1	0	0	1(100)
Vietnam	WPR	LMIC	0	2	2791.5(16.5)2	0	0	2(100)
Zambia	AFR	LMIC	0	1	95(NA)1	1(100)	0	0
Zimbabwe	AFR	LMIC	0	1	4058(NA)1	0	0	1(100)

a Source: World Bank (2024);
b One vaccine could receive its first global approval in multiple countries when the regulatory approval agency is the European Commission.
Abbreviation: AFR, African region. AMR, region of the Americas. EMR, Eastern Mediterranean region. EUR, European region. SEAR, South-East Asia region. WPR, Western Pacific region. HIC, High-income country. LIC, low-income country. LMIC, lower-middle-income country. UMIC, upper-middle-income country. NA, not available.

Table S4 Approval delay of new prophylactic vaccines by indications globally

Indications	Approval delay in days [Median (Interquartile range); N]
Cholera infection	3004 (823); 3
Enterovirus 71 infection	2825 (311); 2
Hepatitis-E virus infection	2015 (NA); 1
Hepatitis-B virus infection	1198 (0); 30
Influenza virus infection	935 (1238); 444
Polio infection	657 (267); 4
Varicella zoster virus infection	397 (382.5); 88
Dengue virus infection	300 (999); 81
Multiple disease	273 (2251.25); 116
Chikungunya virus infection	234 (0); 31
Malaria infection	216 (1652); 3
Meningococcal infection	213 (842); 123
Pneumococcal infection	152 (102); 116
Rotavirus infection	144 (0); 36
Human papilloma virus infection	126 (83); 122
Ebola virus infection	95 (376); 7
Respiratory syncytial virus infection	85 (50); 71
Coronavirus, novel coronavirus infection	69 (138); 529
Japanese encephalitis virus infection	69 (295); 46

Abbreviation: NA, not available.

Table S5: Multivariable negative binomial regression analysis on approval delays estimated by linear regressions with robust standard errors (without COVID-19 vaccines).

**Variable**	Adjusted difference (95%CI)	p value
Income level: HICs as reference		
UMICs	606.44 (344.91 to 867.97)	<0·0001
LMICs or LICs	923.92 (499.29 to 1348.56)	<0·0001
Vaccine valent: Monovalent vaccines as reference		
Multivalent and multipathogen vaccines	215.92 (106.63 to 325.21)	<0·0001
Regulation status of first approval: Usual regulation as reference		
Accelerated regulation	-278.30 (-371.92 to -184.68)	<0·0001
Year of first approval: 2005-2014 as reference		
2015-2024	-143.26 (-244.84 to -41.68)	0.006
manufacturer origin type: small as reference		
Large-size	-236.87 (-398.81 to -74.94)	0.004
Log_2(VPDs incidence cases)	15.37 (-7.36 to 38.10)	0.184

*p<0·01. ‡p<0·05.

Table S6: Multivariable negative binomial regression analysis on approval delays estimated by linear regressions with robust standard errors (using original data).

Variable	Adjusted difference (95%CI)	p value
Income level: HICs as reference		
UMICs	188.40 (64.70 to 312.09)	0.003
LMICs or LICs	313.01(124.77 to 501.26)	0.001
Vaccine valent: Monovalent vaccines as reference		
Multivalent and multipathogen vaccines	142.84 (57.54 to 228.14)	0.001
Regulation status of first approval: Usual regulation as reference		
Accelerated regulation	-290.75 (-380.74 to -200.77)	<0·0001
Year of first approval: 2005-2014 as reference		
2015-2024	-113.16 (-203.76 to -22.55)	0.014
manufacturer origin type: small as reference		
Large-size	3.91 (-10.77 to 18.59)	0.601
Log_2(VPDs incidence cases)	25.92 (-62.60to 114.44)	0.566

*p<0·01. ‡p<0·05.

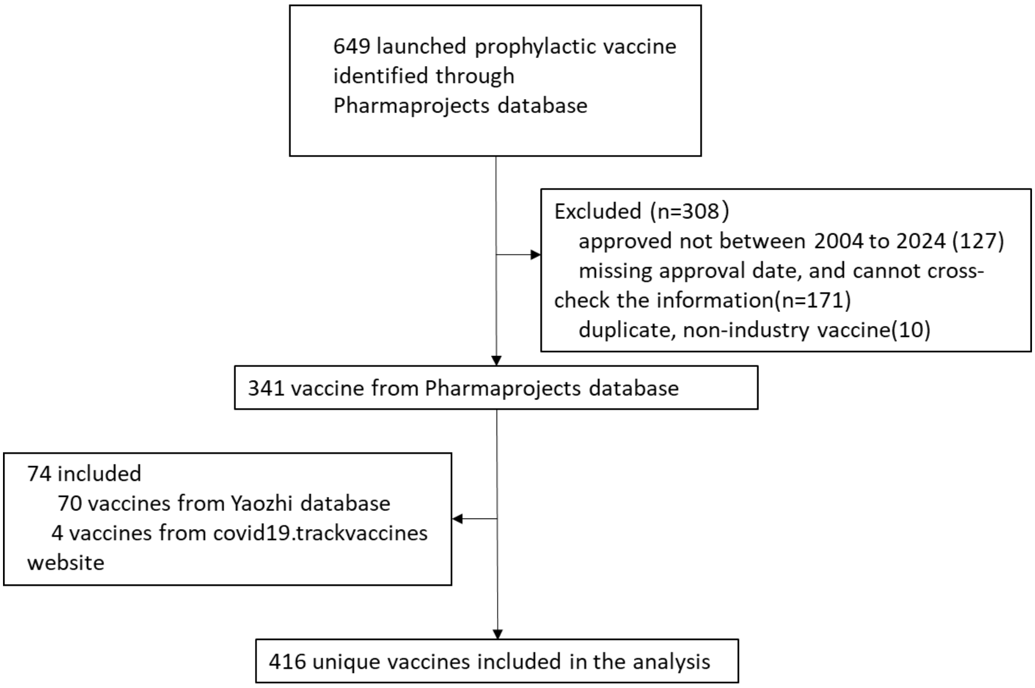

Figure S1 Flow diagram of prophylactic selection from multiple databases

Appendix 2. The STROBE reporting checklist

	Item Description	Location (or reason for not reporting)
**Title and abstract**		
1a. Indicate the study's design	Indicate the study's design with a commonly used term in the title or the abstract.	Abstract
1b. Abstract	Provide in the abstract an informative and balanced summary of what was done and what was found.	Abstract
**Introduction**		
2. Background / rationale	Explain the scientific background and rationale for the investigation being reported.	Introduction
3. Objectives	State specific objectives, including any prespecified hypotheses.	Introduction; paragraph 5
**Methods**		
4. Study design	Present key elements of study design early in the paper.	Data Extraction
5. Setting	Describe the setting, locations, and relevant dates, including periods of recruitment, exposure, follow-up, and data collection.	Data sources and Extraction
6a. Eligibility criteria	**Cohort study:** Give the eligibility criteria, and the sources and methods of selection of participants. Describe methods of follow-up. **Case-control study:** Give the eligibility criteria, and the sources and methods of case ascertainment and control selection. Give the rationale for the choice of cases and controls. **Cross-sectional study:** Give the eligibility criteria, and the sources and methods of selection of participants.	Data Extraction
6b. Matching criteria	**Cohort study:** For matched studies, give matching criteria and number of exposed and unexposed. **Case-control study:** For matched studies, give matching criteria and the number of controls per case.	Not applicable
7. Variables	Clearly define all outcomes, exposures, predictors, potential confounders, and effect modifiers. Give diagnostic criteria, if applicable.	Data Extraction

8. Data sources / measurement	For each variable of interest give sources of data and details of methods of assessment (measurement). Describe comparability of assessment methods if there is more than one group.	Data Extraction & Statistical analysis
9. Bias	Describe any efforts to address potential sources of bias.	Data sources & Statistical analysis
10. Study size	Explain how the study size was arrived at.	Data Extraction
11. Quantitative variables	Explain how quantitative variables were handled in the analyses. If applicable, describe which groupings were chosen, and why.	Statistical analysis
12a. Statistical methods	Describe all statistical methods, including those used to control for confounding.	Statistical analysis
12b. Statistical methods – subgroups and interactions	Describe any methods used to examine subgroups and interactions.	Statistical analysis
12c. Statistical methods – missing data	Explain how missing data were addressed.	Data sources
12di. Statistical methods – loss to follow-up	**Cohort study:** If applicable, describe how loss to follow-up was addressed.	Not applicable
12dii. Statistical methods – matching cases and controls	**Case-control study:** If applicable, explain how matching of cases and controls was addressed.	Not applicable
12diii. Statistical methods – sampling strategy	**Cross-sectional study:** If applicable, describe analytical methods taking account of sampling strategy.	Not applicable
12e. Statistical methods – sensitivity analyses	Describe any sensitivity analyses.	Statistical analysis
**Results**		
13a. Participant numbers	Report the numbers of individuals at each stage of the study—e.g., numbers potentially eligible, examined for eligibility, confirmed eligible, included	Figure S1

	in the study, completing follow-up, and analysed; Consider use of a flow diagram.	
13b. Participants – non-participation	Give reasons for non-participation at each stage.	Figure S1
13c. Participants – flow diagram	Consider use of a flow diagram.	Figure S1
14a. Descriptive data – participant characteristics	Give characteristics of study participants (e.g., demographic, clinical, social) and information on exposures and potential confounders. Present the information in a table.	Table 1
14b. Descriptive data – missing data	Indicate the number of participants with missing data for each variable of interest.	Figure S1
14c. Descriptive data – follow-up time	**Cohort study:** Summarise follow-up time—e.g., average and total amount.	Not applicable
15. Outcome data	**Cohort study:** Report numbers of outcome events or summary measures over time. **Case-control study:** Report numbers in each exposure category, or summary measures of exposure. **Cross-sectional study:** Report numbers of outcome events or summary measures.	Results; paragraph 1
16a. Main results	Give unadjusted estimates and, if applicable, confounder-adjusted estimates and their precision (e.g., 95% confidence intervals). Make clear which confounders were adjusted for and why they were included.	Results; The last paragraph
16b. Main results – category boundaries	Report category boundaries when continuous variables were categorised.	Results; paragraph 1
16c. Main results – risk	If relevant, consider translating estimates of relative risk into absolute risk for a meaningful time period.	Not applicable
17. Other analyses	Report other analyses done—e.g., analyses of subgroups and interactions, and sensitivity analyses.	Results

**Discussion**		
18. Key results	Summarise key results with reference to study objectives.	Discussion; paragraph 1
19. Limitations	Discuss limitations of the study, taking into account sources of potential bias or imprecision. Discuss both direction and magnitude of any potential bias.	Discussion; The last paragraph
20. Interpretation	Give a cautious overall interpretation considering objectives, limitations, multiplicity of analyses, results from similar studies, and other relevant evidence.	Discussion; The last paragraph
21. Generalisability	Discuss the generalisability (external validity) of the study results.	Discussion; The last paragraph
**Other information**		
22. Funding	Give the source of funding and the role of the funders for the present study and, if applicable, for the original study on which the present article is based.	Funding
